# Gene Polymorphisms of Micrornas in *Helicobacter pylori*-Induced High Risk Atrophic Gastritis and Gastric Cancer

**DOI:** 10.1371/journal.pone.0087467

**Published:** 2014-01-27

**Authors:** Juozas Kupcinskas, Thomas Wex, Alexander Link, Marcis Leja, Indre Bruzaite, Ruta Steponaitiene, Simonas Juzenas, Ugne Gyvyte, Audrius Ivanauskas, Guntis Ancans, Vitalija Petrenkiene, Jurgita Skieceviciene, Limas Kupcinskas, Peter Malfertheiner

**Affiliations:** 1 Institute for Digestive Research, Lithuanian University of Health Sciences, Kaunas, Lithuania; 2 Department of Gastroenterology, Lithuanian University of Health Sciences, Kaunas, Lithuania; 3 Department of Gastroenterology, Hepatology and Infectious Diseases, Otto von Guericke University, Magdeburg, Germany; 4 Medical Laboratory for Clinical Chemistry, Microbiology and Infectious Diseases, Department of Molecular Genetics, Magdeburg, Germany; 5 Faculty of Medicine, University of Latvia, Riga, Latvia; 6 Digestive Diseases Center GASTRO, Riga, Latvia; 7 Riga East University Hospital, Riga, Latvia; National Cancer Center, Japan

## Abstract

**Background and aims:**

MicroRNAs (miRNAs) are known for their function as translational regulators of tumor suppressor or oncogenes. Single nucleotide polymorphisms (SNPs) in miRNAs related genes have been shown to affect the regulatory capacity of miRNAs and were linked with gastric cancer (GC) and premalignant gastric conditions. The purpose of this study was to evaluate potential associations between miRNA-related gene polymorphisms (*miR-27a*, *miR-146a*, *miR-196a-2*, *miR-492* and *miR-608*) and the presence of GC or high risk atrophic gastritis (HRAG) in European population.

**Methods:**

Gene polymorphisms were analyzed in 995 subjects (controls: n = 351; GC: n = 363; HRAG: n = 281) of European descent. *MiR-27a* T>C (rs895819), *miR-146a* G>C (rs2910164), *miR-196a-2* C>T (rs11614913), *miR-492* G>C (rs2289030) and *miR-608* C>G (rs4919510) SNPs were genotyped by RT-PCR.

**Results:**

Overall, SNPs of miRNAs were not associated with the presence of GC or HRAG. We observed a tendency for *miR-196a-2* CT genotype to be associated with higher risk of GC when compared to CC genotype, however, the difference did not reach the adjusted *P*-value (odds ratio (OR) - 1.46, 95% confidence interval (CI) 1.03-2.07, *P* = 0.032). *MiR-608* GG genotype was more frequent in GC when compared to controls (OR −2.34, 95% CI 1.08–5.04), but significance remained marginal (*P* = 0.029). A similar tendency was observed in a recessive model for *miR-608*, where CC + CG vs GG genotype comparison showed a tendency for increased risk of GC with OR of 2.44 (95% CI 1.14–5.22, *P* = 0.021). The genotypes and alleles of *miR-27a*, *miR-146a*, *miR-196a-2*, *miR-492* and *miR-608* SNPs had similar distribution between histological subtypes of GC and were not linked with the presence of diffuse or intestinal-type GC.

**Conclusions:**

Gene polymorphisms of *miR-27a*, *miR-146a*, *miR-196a-2*, *miR-492*, *miR-492a* and *miR-608* were not associated with the presence of HRAG, GC or different histological subtypes of GC in European subjects.

## Introduction

Despite decreasing incidence of gastric cancer (GC) in most developed countries, GC accounted for a total of 989,600 new cases and 738,000 deaths in 2008 worldwide [1]. *Helicobacter pylori* (*H. pylori*) infection in gastric mucosa may lead to the development of atrophic gastritis (AG) and intestinal metaplasia (IM) and is a cardinal risk factor for development of GC [2]. Nevertheless, the development of gastric adenocarcinoma cannot be explained by the presence of *H. pylori* alone. Synergistic effects of environmental, host, nutritional and bacterial factors are believed to trigger gastric carcinogenesis, however, the mechanisms and interaction are still poorly understood [3]. A number of gene alterations have been implicated in gastric carcinogenesis, but none of them has been yet transferred to daily clinical practice due to the lack of association strength [4]. Recent data suggest a potential influence of single nucleotide polymorphisms (SNPs) of microRNA-related genes (miRNAs) for the risk of cancer development [5].

MiRNAs are only ∼22 bp long, and because of its unique biogenesis are highly stable in different tissues or specimens, making them an attractive target in biomarker research field [6], [7]. A growing number of functional studies suggest that miRNAs may be involved in different stages of gastric carcinogenesis [8]. This concordance is also reflected in miRNA profiling studies that revealed specific alterations in expression pattern in mucosa of GC patients. Furthermore, miRNA expression changes are already detectable in early stages of gastric carcinogenesis including *H. pylori* induced AG [8].

Increasing understanding of functional role of miRNAs has opened a new capitel in gene polymorphism research in cancer [7], [9]. Single miRNA is capable of targeting multiple genes; therefore, the significance of the SNPs in miRNA gene sequence may potentially be associated with remarkable alterations in regulation of gene expression and modification of the risk to development of certain human diseases, including GC [8]. Different studies have shown that certain SNPs of miRNA-encoding genes may alter miRNA expression or its functional role and in this way influence the risk of cancer development or progression [10], [11]. Growing number of case-control studies have shown association between the polymorphisms of the genes encoding miRNAs and the risk of different malignancies [5]. Gene polymorphisms of five miRNA-related genes, *miR-27a* T>C (rs895819), *miR-146a* G>C (rs2910164), *miR-196a-2* C>T (rs11614913), *miR-492* G>C (rs2289030) and *miR-608* C>G (rs4919510), have been chosen for the present study because of previously suggested association with cancer risks [5], [12]–[14].

The above mentioned miRNAs have been implicated in various cancer-related pathways. Liu et al. showed that *miR-27a* is up-regulated in GC and acts as an oncogene by targeting prohibitin [15]. SNP of *miR-27a* (rs895819) contributes to GC susceptibility through affecting the expression of *miR-27a* and targets gene zinc finger and BTB domain containing 10 (*ZBTB10*) [16]. *MiR-146a* was shown to modulate *H. pylori* induced inflammatory response in human gastric epithelial cells by targeting IL-1 associated kinase 1 (*IRAK1*) and TNF receptor associated factor 6 (*TRAF6*) [17]. Furthermore, *miR-146a* SNP (rs2910164) has been associated with AG in Japanese subjects [18]. Previous studies have demonstrated aberrant over expression of *miR-196a-2* and consequent down regulation of p27 (kip1) in GC [16]. Another study has shown that gene polymorphism of *miR-196a-2* (rs11614913) was associated with increased risk for GC [19], [20] while a study by Ahn et al. (2012) could not confirm this association, but this SNP was linked with survival in GC patients [21]. *MiR-492* was identified to play an important role in the progression of malignant embryonic liver tumors [22] and is deregulated in colorectal cancer when compared to normal colon mucosa [23]. A report in Chinese population showed that SNP of *miR-608* (rs4919510) may influence HER2-positive breast cancer risk and tumor proliferation [14].

Most of the currently published genotyping studies related to miRNA genes in GC were performed only in Asian subjects, are limited by small sample sizes and report partially conflicting results. Furthermore, very few studies have previously addressed the role of these miRNA-related SNPs in premalignant gastric conditions. Based on the evidence provided above, we performed systematic genotyping analysis for *miR-27a*, *miR-146a*, *miR-196a-2*, *miR-492* and *miR-608* SNPs in patients with GC, high risk atrophic gastritis and controls using three groups of patients from Germany, Lithuania and Latvia of European descent.

## Methods

### Ethics statement

The study was approved by the Ethics Committees of the Otto-von-Guericke University Magdeburg, Lithuanian University of Health Sciences and Central Medical Ethics Committee of of Latvia. All patients have signed an informed consent form to participate in the study.

### Study population

Subjects included in the study come from our previous research projects on SNPs in GC and premalignant gastric conditions [24], [25]. Patients and controls were recruited during the years 2005–2012 at three gastroenterology centers in Germany (Department of Gastroenterology, Hepatology and Infectious Diseases, Otto-von-Guericke University, Magdeburg) Lithuania (Department of Gastroenterology, Lithuanian University of Health Sciences, Kaunas) and Latvia (Riga East University Hospital and Digestive Diseases Centre GASTRO, Riga). Patients with HRAG and controls were included from the out-patient departments, who were referred for upper endoscopy because of dyspeptic symptoms. The inclusion criteria of HRAG and controls were no history of malignancy, gastrointestinal disease or surgery. GC patients had histological verification of gastric adenocarcinoma and were recruited from out-patient and stationary departments. In total, 995 individuals, for whom material with appropriate DNA quality was available (351 controls, 281 HRAG and 363 GC), were included in the study. There were 310 subjects from German group (63 controls, 106 GC and 141 HRAG), 340 subjects from Latvian group (142 controls, 139 GC and 59 HRAG) and 345 subjects from Lithuanian group (146 controls, 118 GC and 81 HRAG). All patients were of European descent.

### Histological analysis and *H. pylori* status

Detailed histological evaluation of gastric mucosa was performed in controls and HRAG groups according to the modified Sydney classification [26]. HRAG was defined as pan-gastritis (similar inflammatory scores in antrum and corpus), corpus-predominant gastritis with or without the presence of gastric atrophy, and IM either in antrum or corpus of the stomach [27]. *H. pylori* status was determined by testing for anti-*H. pylori* IgG antibodies in sera. Histological subtyping of GC was carried out according to the Laurén classification into intestinal and diffuse-types [28].

### DNA extraction and genotyping

Genomic DNA from German group was extracted from peripheral blood mononuclear cells using the QIAamp DNA blood kit (Qiagen, Hilden, Germany) according to the manufacturer's instructions. Genomic DNA from samples of Lithuanian and Latvian groups was extracted using phenol-chloroform extraction method from peripheral blood mononuclear cells. DNA samples were stored at −20°C until analysis. SNPs of *miR-27a* C>G (rs895819), *miR-146a* C>G (rs2910164), *miR-196a-2* C>T (rs11614913), *miR-492* C>G (rs2289030) and *miR-608* C>G (rs4919510) were genotyped by using predesigned TaqMan® assays with a 7500™ real-time cycler, in accordance with the manufacturer's instructions (Life Technologies, CA, USA). Genotype assignments were manually confirmed by visual inspection with the SDS 2.0.5 software compatible with the TaqMan® system.

### Genotyping quality control

Quality control procedures were applied throughout genotyping of the samples. After genotyping, approximately 10% of samples in each genotype group were selected for repetitive analysis with 100% concordance rate. Dubious samples had triplet repetitive analysis. All parties involved in genotyping were blinded to the case or control status of the samples. Samples that failed to genotype were recorded as undetermined.

### Statistical analysis

All subjects were classified into three study groups: controls (n = 351), HRAG (n = 281), GC (n = 363). Age is shown as means and standard deviations, and was compared using ANOVA and unpaired Student's t-test. Categorical data (e.g. gender, *H. pylori* infection, histological GC subtypes, distribution of genotypes or alleles) are presented as frequencies; comparisons were performed using the Chi-square test.

Quality assessments and statistical analysis of the genotyping data were performed using PLINK software version 1.07 [29]. Individuals with more than 10% missing genotypes and SNPs with a call-rate below 90% or deviation from Hardy-Weinberg equilibrium (HWE) in the controls (*P*<0.05) were excluded from further analysis. The average genotyping rate across all samples was 99.6%. Differences in allele frequencies between cases and controls were calculated in the combined German, Lithuanian and Latvian study sample, using the Breslow-Day test for heterogeneity of ORs. Only one SNP (*miR-608* C>G rs4919510) showed heterogeneity of ORs between the three GC study groups (*P*_BD_<0.05) and, therefore, the country of birth was included as the covariate in further analysis. Association between HRAG and GC with gene polymorphisms was calculated using logistic regression analysis with adjustment for age, gender and country of birth with 95% confidence intervals (CI). The relative risks for mutations were studied using recessive and dominant model that led to a comparison between wild type + heterozygous vs. homozygous and wild type *vs.* heterozygous + homozygous, respectively. To adjust for multiple testing we calculated a corrected significance threshold α = 0.01 (0.05/5).

## Results

### Characteristics of the subjects

The characteristics of control, GC and HRAG groups are presented in **Table 1**. In accordance with real-life age, subjects differed significantly according to age and gender distribution between the groups. As expected, males accounted for 63.6% in GC group, while in control and HRAG groups this gender constituted 26.8% and 37.7%, respectively. Control subjects were significantly younger than HRAG group (3 years) and GC group (5 years). 52.6% of patients were positive for *H. pylori* in GC group, however, in around 27% of individuals in GC group *H. pylori* IgG status could not be obtained. Over 47% of subjects in control group and 42.7% in HRAG group were *H. pylori* positive. Histological classification for diffuse and intestinal GC types was retrieved for 62.0% of GC patients (**Table 1**).

**Table 1:** Characteristics of subject groups.

	Controls (n = 351)	GC (n = 363)^1^	HRAG (n = 281)^1^	ANOVA (Age)^2^ Chi-squared test *P* value
Age				
Mean ± SD	60.4±13.9	65.3±12.5	63.3±10.4	<0.001
				
Gender				
Male	94 (26.8%)	231 (63.6%)	106 (37.7%)	<0.001
Female	257 (73.2%)	132 (36.4%)	175 (62.3%)	
*H. pylori* 1				
Positive	166 (47.3%)	191 (52.6%)	120 (42.7%)	<0.001
Negative	184 (52.4%)	74 (20.4%)	161 (57.3%)	
Unknown	1 (0.3%)	98 (27.0%)		
GC Lauren type^1^				
Intestinal		136 (37.5)		
Diffuse		89 (24.5)		
Mixed		33 (9.1)		
Data unavailable		105 (28.9)		

1 GC – gastric cancer; HRAG – high risk gastritis; *H. pylori* – *Helicobacter pylori.*

2 Statistical analysis was performed globally for all three groups.

### Associations of miRNA SNPs and risk of GC and HRAG

Genotype distribution for all five polymorphisms in the study were similar to those expected for Hardy-Weinberg Equilibrium: rs895819 (p = 0.94); rs2910164 (p = 0.44); rs11614913 (p = 0.95); rs2289030 (p = 0.44); rs4919510 (p = 0.39). Genotype and allele distributions for *miR-27a* C>G (rs895819), *miR-146a* C>G (rs2910164), *miR-196a-2* C>T (rs11614913), *miR-492* C>G (rs2289030) and *miR-608* C>G (rs4919510) gene polymorphisms in GC and HRAG study groups are presented in **Table 2**. No significant associations were observed for diseases under study following correction for multiple testing. We observed a tendency for *miR-196a-2* CT genotype to be associated with higher risk of GC when compared to CC genotype, however, the difference did not reach the adjusted significance threshold (OR - 1.46, 95% CI 1.03–2.07, *P* = 0.032). *MiR-608* GG genotype was marginally associated with higher risk of GC when compared to CC genotype (OR - 2.34, 95% CI 1.08–5.04, *P* = 0.029). A similar tendency was observed in a recessive model for *miR-608*, where CC + CG vs GG genotype comparison resulted in an OR - 2.44 (95% CI 1.14–5.22), (*P* = 0.021).

**Table 2:** Genotype frequencies of *miR-27a*, *miR-146a*, *miR-196a-2*, *miR-492*, *miR-608* SNPs in controls, gastric cancer and high risk gastritis patients.

Genotype	Controls (n = 351)	GC^1^ (n = 363)			HRAG^1^ (n = 281)		
	n (%)	n (%)	aOR (95% CI)^1^	*P*	n (%)	aOR (95% CI)^1^	*P*
***miR-27a*** ** (rs895819)** 2							
TT	156 (44.6)	181 (49.9)	1 (Reference)		129 (46.7)	1 (Reference)	
TC	164 (46.9)	144 (39.7)	0.73 (0.52–1.03)	0.080	120 (43.5)	1.01 (0.61–1.67)	0.959
CC	30 (8.6)	38 (10.5)	0.97 (0.54–1.77)	0.944	27 (9.8)	3.52 (0.35–34.7)	0.280
TT vs TC + CC			0.77 (0.55–1.07)	0.123		1.07 (0.66–1.75)	0.765
TT + TC vs. CC			1.13 (0.64–2.00)	0.660		3.52 (0.35–34.6)	0.280
Allele T	476 (68.0)	506 (69.7)	0.88 (0.68–1.13)	0.318	387 (69.0)	1.13 (0.87–1.37)	0.462
Allele C	224 (32.0)	220 (30.3)			174 (31.0)		
***miR-146a*** ** (rs2910164)** 3							
GG	223 (64.3)	252 (69.6)	1 (Reference)		170 (60.5)	1 (Reference)	
GC	108 (31.1)	94 (26.0)	0.68 (0.47–0.99)	0.047	101 (35.9)	1.08 (0.76–1.53)	0.646
CC	16 (4.6)	16 (4.4)	0.87 (0.40–1.91)	0.742	10 (3.6)	1.18 (0.73–1.93)	0.484
GG vs GC +. CC			0.71 (0.50–1.01)	0.058		1.11 (0.80–1.53)	0.524
GG + GC vs CC			0.98 (0.45–2.11)	0.968		0.77 (0.34–1.74)	0.536
Allele G	554 (79.8)	598 (82.6)	0.79 (0.60–1.06)	0.119	441 (78.5)	1.08 (0.86–1.36)	0.462
Allele C	140 (20.2)	126 (17.4)			121 (21.5)		
***miR-196a-2*** **(rs11614913)** 4							
CC	159 (45.4)	144 (39.7)	1 (Reference)		121 (43.1)	1 (Reference)	
CT	145 (41.4)	184 (50.7)	1.46 (1.03–2.07)	0.032	118 (42.0)	1.22 (0.86–1.72)	0.256
TT	46 (13.1)	35 (9.6)	0.95 (0.55–1.63)	0.851	42 (14.9)	0.83 (0.36–1.89)	0.660
CC vs. CT + TT			1.34 (0.96–1.87)	0.083		1.17 (0.84–1.63)	0.351
CC + CT vs. TT			0.78 (0.47–1.30)	0.339		0.77 (0.34–1.74)	0.536
Allele C	463 (66.1)	472 (65.0)	1.11 (0.87–1.41)	0.412	360 (64.1)	1.08 (0.82–1.44)	0.567
Allele T	237 (33.9)	254 (35.0)			202 (35.9)		
***miR-492*** ** (rs2289030)**							
GG	310 (88.3)	312 (86.0)	1 (Reference)		246 (87.5)	1 (Reference)	
GC	40 (11.4)	49 (13.5)	1.19 (0.72–1.95)	0.492	32 (11.4)	0.85 (0.60–1.19)	0.353
CC	1 (0.3)	2 (0.6)	2.99 (0.21–41.1)	0.411	3 (1.1)	1.11 (0.62–1.98)	0.718
GG vs. GC + CC			1.22 (0.74–2.00)	0.416		0.89 (0.64–1.23)	0.485
GG + GC vs.CC			2.93 (0.21–40.2)	0.420		1.20 (0.69–2.09)	0.512
Allele G	660 (94.0)	673 (92.7)	1.25 (0.78–1.99)	0.356	524 (93.2)	0.97 (0.76–1.24)	0.804
Allele C	42 (6.0)	53 (7.3)			38 (6.8)		
***miR-608*** ** (rs4919510)** 5							
CC	251 (71.7)	250 (68.9)	1 (Reference)		197 (70.6)	1 (Reference)	
GC	86 (24.6)	88 (24.2)	0.84 (0.57–1.24)	0.395	74 (26.5)	1.07 (0.73–1.54)	0.719
GG	13 (3.7)	25 (6.9)	2.34 (1.08–5.04)	0.029	8 (2.9)	0.79 (0.32–1.98)	0.627
CC vs. CG + GG			1.01 (0.70–1.45)	0.938		1.03 (0.72–1.47)	0.851
CC + CG vs. GG			2.44 (1.14–5.22)	0.021		0.78 (0.31–1.94)	0.598
Allele C	588 (84.0)	588 (81.0)	1.15 (0.87–1.53)	0.334	468 (83.9)	0.99 (0.74–1.35)	0.985
Allele G	112 (16.0)	138 (19.0)			90 (16.1)		

1 GC – gastric cancer; HRAG – high risk gastritis; aOR – adjusted odds ratio (age, sex, country); CI – confidence interval.

2 six patients with missing values on rs895819 were excluded from analysis.

3 five patients with missing values on rs2910164 were excluded from analysis.

4 one patient with missing values on rs11614913 was excluded from analysis.

5 three patients with missing values on rs4919510 were excluded from analysis.

### Associations of miRNA SNPs and risk of intestinal and diffuse-type GC

*MiR-27a*, *miR-146a*, *miR-196a-2*, *miR-492* and *miR-608* SNP associations were tested with two histological types of GC (diffuse or intestinal type-GC). Genotype and allele frequencies for the study groups are presented in **Table 3** **.** Dominant model for *miR-27a* showed a tendency for allele T vs. C to be linked with higher risk of diffuse-type GC (OR - 2.07, 95% CI 1.13–3.79, *P* = 0.018). *MiR-196-a2* SNP showed a tendency towards reduced risk of intestinal type GC in recessive model (CC + CT vs. TT), however, the association strength did not reach statistical significance (OR – 0.47, 95% CI 0.20–1.08, *P* = 0.077). Similar observation was made in a dominant model for *miR-492* SNP (OR - 1.95, 95% CI 1.01–3.75) where GG vs. GC comparison led to an increased risk of diffuse-type GC, however the *P* value did not reach required significance level (*P* = 0.046). All the other comparisons between control and diffuse or intestinal-type GC groups did not reveal significant associations or trends for five SNPs of miRNAs.

**Table 3:** Genotype frequencies of *miR-27a*, *miR-146a*, *miR-196a-2*, *miR-492*, *miR-608* SNPs in controls, intestinal and diffuse-type GC subjects.

Genotype	Controls (n = 351)	Intestinal GC^1^ (n = 136)			Diffuse GC^1^ (n = 89)		
	n (%)	n (%)	aOR (95% CI)^1^	*P*	n (%)	aOR (95% CI)^1^	*P*
***miR-27a*** ** (rs895819)**							
TT	156 (44.6)	70 (51.9)	1 (Reference)		46 (51.7)	1 (Reference)	
TC	164 (46.9)	52 (38.5)	0.64 (0.40–1.02)	0.065	39 (43.8)	0.78 (0.47–1.29)	0.343
CC	30 (8.6)	13 (9.6)	0.89 (0.40–1.97)	0.780	4 (4.5)	0.44 (0.14–1.38)	0.163
TT vs. TC + CC			0.68 (0.44–1.06)	0.090		0.73 (0.45–1.19)	0.210
TT + TC vs. CC			1.10 (0.51–2.35)	0.804		0.50 (0.16–1.51)	0.223
Allele T	476 (68.0)	192 (71.1)	1.24 (0.67–2.29)	0.495	141 (75.0)	2.07 (1.13–3.79)	0.018
Allele C	224 (32.0)	78 (28.9)			47 (25.0)		
***miR-146a*** ** (rs2910164)**							
GG	223 (64.3)	88 (65.2)	1 (Reference)		64 (71.9)	1 (Reference)	
GC	108 (31.1)	43 (31.9)	0.95 (0.59–1.53)	0.841	21 (23.6)	0.65 (0.37–1.15)	0.143
CC	16 (4.6)	4 (3.0)	0.77 (0.24–2.49)	0.673	4 (4.5)	0.83 (0.26–2.66)	0.760
GG vs GC +. CC			0.93 (0.58–1.47)	0.761		0.67 (0.39–1.15)	0.154
GG + GC vs CC			0.79 (0.24–2.51)	0.690		0.95 (0.30–3.00)	0.932
Allele G	554 (79.8)	219 (81.1)	0.99 (0.71–1.38)	0.945	149 (83.7)	1.09 (0.77–1.54)	0.618
Allele C	140 (20.2)	51 (18.9)			29 (16.3)		
**miR-196a2 (rs11614913)**							
CC	159 (45.4)	57 (41.9)	1 (Reference)		37 (41.6)	1 (Reference)	
CT	145 (41.4)	71 (52.2)	1.46 (0.92–2.32)	0.103	41 (46.1)	1.29 (0.76–2.18)	0.332
TT	46 (13.1)	8 (5.9)	0.57 (0.24–1.36)	0.211	11 (12.4)	1.05 (0.48–2.28)	0.898
CC vs. CT + TT			1.25 (0.80–1.95)	0.312		1.23 (0.75–2.01)	0.403
CC + CT vs. TT			0.47 (0.20–1.08)	0.077		0.92 (0.44–1.91)	0.835
Allele C	463 (66.1)	185 (68.0)	0.92 (0.63–1.37)	0.692	115 (64.6)	0.76 (0.48–1.19)	0.229
Allele T	237 (33.9)	87 (32.0)			63 (35.4)		
***miR-492*** ** (rs2289030)**							
GG	310 (88.3)	114 (83.8)	1 (Reference)		71 (79.8)	1 (Reference)	
GC	40 (11.4)	22 (16.2)	1.28 (0.68–2.42)	0.428	17 (19.1)	1.95 (1.01–3.75)	0.046
CC	1 (0.3)	0 (0.0)	0.36 (0.09–0.54)	0.999	1 (1.1)	8.01 (0.48–132.1)	0.145
GG vs. GC + CC			1.26 (0.67–2.37)	0.456		2.05 (1.08–3.91)	0.028
GG + GC vs. CC			0.35 (0.07–0.68)	0.999		7.21 (0.43–118.6)	0.166
Allele G	660 (94.0)	250 (91.9)	0.81 (0.57–1.14)	0.226	159 (89.3)	0.73 (0.49–1.09)	0.128
Allele C	42 (6.0)	22 (8.1)			19 (10.7)		
***miR-608*** ** (rs4919510)**							
CC	251 (71.7)	96 (70.6)	1 (Reference)		63 (70.8)	1 (Reference)	
GC	86 (24.6)	31 (22.8)	0.74 (0.43–1.26)	0.279	25 (28.1)	0.98 (0.56–1.72)	0.950
GG	13 (3.7)	9 (6.6)	2.03 (0.74–5.55)	0.164	1 (1.1)	0.44 (0.05–3.55)	0.447
CC vs. CG + GG			0.88 (0.54–1.44)	0.624		0.92 (0.53–1.60)	0.788
CC + CG vs. GG			2.18 (0.81–5.89)	0.122		0.44 (0.05–3.55)	0.448
Allele C	588 (84.0)	223 (82.0)	1.04 (0.70–1.53)	0.858	151 (84.8)	0.89 (0.55–1.44)	0.637
Allele G	112 (16.0)	49 (18.0)			27 (15.2)		

1 GC – gastric cancer; HRAG – high risk gastritis; aOR – adjusted odds ratio (age, sex, country); CI – confidence interval.

## Discussion

In the present study we performed a genotyping analysis for *miR-27a* (rs895819), *miR-146a* (rs2910164), *miR-196a-2* (rs11614913), *miR-492* (rs2289030) and *miR-608* (rs4919510) gene polymorphisms in a case control study including 351 controls, 281 HRAG and 363 GC patients from three European study groups. These polymorphisms have been associated with the risk of GC or overall cancer risks; however, reported data are partially conflicting or based on only few studies related to Asian groups. Despite some minor differences, our results do not support the link between *miR-27a*, *miR-146a*, *miR-196a-2*, *miR-492* and *miR-608* gene polymorphisms and the presence of GC or HRAG. Furthermore, we could not find an association between SNPs of miRNAs and the presence of different histological subtypes of GC. Since miRNAs have been shown to have profound effect in gastric carcinogenesis [8], we expected that these polymorphisms could modify the risk of GC or its precursors. To our best knowledge, this is the first study which investigated the associations between these miRNA polymorphisms and the risk of GC or HRAG in subjects of European descent. More than that, SNPs of *miR-492* (rs2289030) and *miR-608* (rs4919510) have not been studied in relation to GC or premalignant gastric conditions previously.

Gene polymorphism of miR-27a is at present one of the most studied SNPs in cancer related case-control studies. We expected that carriers of C allele for *miR-27a* SNP could have a reduced risk of GC as has been suggested by some research groups. A Chinese case-control study including 311 GC patients and 425 cancer-free controls found that minor allele C of rs895819 in the *miR-27a* significantly reduced risk of GC with OR - 0.77 [30]. Another study showed a significantly increased risk of GC (OR - 1.48) for *miR-27a* SNP and further functional analyses indicated that variant genotypes might be responsible for elevated *miR-27a* levels and reduced *ZBTB10* mRNA levels [16]. In contrast, a study including 2,380 participants with diverse gastric lesions did not find an increased risk of gastric IM or dysplasia for rs895819 [12]. A meta-analysis summarizing the findings of different case-control studies revealed that rs895819 polymorphism was significantly associated with decreased risks of cancer in white, but not in Asians [31]. The results of our study do not support the possible protective effect of C allele for GC development in European subjects. Our study is the first which evaluated the role of *miR-27a* rs895819 in GC and HRAG in subjects of European descent, however, no significant associations have been observed.

Considering the existing evidence on *miR-146a* SNP (rs2910164) for cancer development we wanted to assess the possible association in our group of patients with GC and HRAG. Song et al. found that CC carriers of rs2910164 had a significantly increased risk of IM (OR - 1.42) and dysplasia (OR - 1.54) compared to GG carriers [12]. A similar observation was made in another study where combined effect of *miR-146a* (rs2910164) G/G and TLR4 SNP on the increased risk of severe AG among Japanese subjects was observed [18]. A paper by Okubo et al. [32] reported that carriage of rs2910164 CC genotype was associated with a significantly higher risk of GC when compared to non-cancer subjects (OR - 1.30). A meta-analysis by Wang et al. [33] on *miR-146a* rs2910164 including 19 case-control studies found that rs2910164 was linked with increased cancer susceptibility in Asians with overall OR - 1.18, but the same meta-analysis urges for further well-designed studies with large sample size for further risk identification. The results of our case-control study did not reveal a significant association between rs2910164 and the risk of GC or HRAG.

*MiR-196a-2* C>T SNP (rs11614913) is one of the best studied miRNA SNPs in relation to different malignancies. A report by Wang et al. (2013) showed that CC genotype was associated with a significantly reduced risk of gastric cancer (OR - 0.78) compared with the CT and TT genotypes in a large case-control study [33]. A meta-analysis by Wang et al. (2013) found a significantly increased GC risk, but the association was observed only in homozygote comparison. This meta-analysis demonstrated that rs11614913 polymorphism is significantly associated with overall risk of gastrointestinal cancers [34]. Okubo et al. [32] did not find a link for this SNP and GC, however, in their study rs11614913 was associated with the degree of *H. pylori*-induced mononuclear cell infiltration. Interestingly, another meta-analysis on the same SNP did not determine relationship between rs11614913 and GC development [35]. The results of our study are in line with the conclusion of the latter meta-analysis as we did not observe significant association between SNP of *miR-196a-2* and GC risk.

Polymorphism of *miR-492* (rs2289030) is hypothesized to mediate cancer risk, but the data on possible associations for this SNP and cancer risk are still scarce. A study by Yoon et al. (2012) did not find an association between SNP of *miR-492* and the risk or survival in patients with non-small cell lung cancer [36]. Another study on colorectal cancer has demonstrated that progression-free survival of the patients with the combined *miR-492* CG and GG genotype was significantly worse than that of the patients with the *miR-492* CC genotype [13]. Our results indicate that rs2289030 of *miR-492* was not linked with the presence of GC or HRAG. To our best knowledge this is the first study which examines the relationship between *miR-492* SNP and premalignant gastric conditions or GC and, therefore, no comparison for our data can made.

SNP of *miR-608* (rs4919510) was not studied in relation to GC risks previously. Our study is the first to report null association between this polymorphism and the risk of GC or premalignant gastric conditions. In colorectal cancer patients rs4919510 was significantly linked with cancer recurrence and death [37]. Another case-control study on colorectal cancer patients did not find an association with cancer risk, but their results showed that GG genotype was associated with an increased risk of death in white population and reduced risk of death in African Americans [38]. Further larger scale studies might determine the role *miR-608* rs4919510 SNP in gastric and other malignancies.

Intestinal and diffuse-types of GC have been shown to have different pathogenetic pathways [3]. Some studies have found that miRNA polymorphisms might have different effects for histological subtypes of GC. After stratifying the patients in intestinal and diffuse-type GC groups, *miR-499* rs3746444 was shown to affect the risk of diffuse-type GC in Korean population [21]. Although the number of individuals within intestinal and diffuse-type GC groups was not very high in our study, analysis comparing these two distinct histological types of GC was performed. Overall, *miR-27a*, *miR-146a*, *miR-196a-2*, *miR-492* and *miR-608* SNPs were not linked with the presence of different histological subtypes of GC. Although some tendencies described in the results section have been observed between the histological subgroup of GC, the differences in genotype and allele frequencies did not reach required significance levels.

Results of our study clearly support the importance of careful evaluation of gene polymorphisms in different ethnical groups. Previously suggested miRNA-related SNPs did not show substantial GC related differences in our European population. Nevertheless, we admit that there are several limitations related to the design of our study. There were gender distribution differences between GC, HRAG and control groups; however, when performing statistical analysis we included gender as a covariate, thus minimizing the potential influence of gender for the outcome of our results. Here in this study we focused on histological changes rather than the presence of *H. pylori*; therefore, additional tests apart from serum IgG antibodies for detection this bacterium were not applied. Furthermore, it is well know that chronic atrophic gastritis is caused by *H. pylori* infection. We did not adjust OR for *H. pylori* IgG antibodies, *CagA* or *VacA* status because this information was not available for all the subjects. We could not perform association analysis for SNPs of miRNAs with respect to survival of GC patients, as the information was available only for a small proportion of subjects. Due to the same reason we also did not perform analysis according to the anatomic location of the in the stomach (proximal vs. distal). In this study we performed solely gene polymorphism analysis without addressing the miRNA expression differences in cancerous and non-cancerous tissues and, therefore, we cannot postulate if these polymorphisms could have a functional role. Further studies should evaluate the role of SNPs for miRNA levels in GC tissue and assess potential target genes in functional studies. The number of individuals within the subgroups in our study of intestinal and diffuse-type GC groups is relatively small and might be underpowered to detect specific associations. We found some differences for miR-196a-2 and miR-608 SNPs among the groups with *P*<0.05; however, we think that we cannot report a significant association, since they did not reach our adjusted *P* value. Multiple comparisons for genetic association tests have been applied in this paper and we believe that we should use the adjusted *P* value for drawing the conclusions.

## Conclusions

Our study shows that gene polymorphisms of *miR-27a*, *miR-146a*, *miR-196a-2*, *miR-492* and *miR-608* are not associated with risk of GC and HRAG in subjects of European descent. These SNPs do not appear as potential biomarkers for identifying individuals with increased risk for GC.

## References

[1] Jemal A, Bray F (2011) Center MM, Ferlay J, Ward E, et al (2011) Global cancer statistics. CA Cancer J Clin 61: 69–90. 21296855 10.3322/caac.20107

[2] Correa P (1992) Human gastric carcinogenesis: A multistep and multifactorial process—first american cancer society award lecture on cancer epidemiology and prevention. Cancer Res 52: 6735–6740. 1458460

[3] Bornschein J, Kandulski A, Selgrad M, Malfertheiner P (2010) From gastric inflammation to gastric cancer. Dig Dis 28: 609–614. 21088411 10.1159/000320061

[4] Malfertheiner P, Megraud F, O'Morain CA, Atherton J, Axon AT, et al (2012) Management of *Helicobacter pylori* infection—the Maastricht IV/Florence consensus report. Gut 61: 646–664. 22491499 10.1136/gutjnl-2012-302084

[5] Srivastava K, Srivastava A (2012) Comprehensive review of genetic association studies and meta-analyses on miRNA polymorphisms and cancer risk. PLoS One 7: e50966. 23226435 10.1371/journal.pone.0050966 PMC3511416

[6] Calin GA, Croce CM (2006) MicroRNA signatures in human cancers. Nat Rev Cancer 6: 857–866. 17060945 10.1038/nrc1997

[7] Mishra PJ, Bertino JR (2009) MicroRNA polymorphisms: The future of pharmacogenomics, molecular epidemiology and individualized medicine. Pharmacogenomics 10: 399–416. 19290790 10.2217/14622416.10.3.399 PMC2705205

[8] Link A, Kupcinskas J, Wex T, Malfertheiner P (2012) Macro-role of microRNA in gastric cancer. Dig Dis 30: 255–267. 22722550 10.1159/000336919

[9] Ryan BM, Robles AI, Harris CC (2010) Genetic variation in microRNA networks: The implications for cancer research. Nat Rev Cancer 10: 389–402. 20495573 10.1038/nrc2867 PMC2950312

[10] Jazdzewski K, Murray EL, Franssila K, Jarzab B, Schoenberg DR, et al (2008) Common SNP in pre-miR-146a decreases mature miR expression and predisposes to papillary thyroid carcinoma. Proc Natl Acad Sci U S A 105: 7269–7274. 18474871 10.1073/pnas.0802682105 PMC2438239

[11] Yang Q, Jie Z, Ye S, Li Z, Han Z, et al.. (2012) Genetic variations in miR-27a gene decrease mature miR-27a level and reduce gastric cancer susceptibility. Oncogene. Epub ahead of print. 10.1038/onc.2012.569 23246964

[12] Song MY, Su HJ, Zhang L, Ma JL, Li JY, et al (2013) Genetic polymorphisms of miR-146a and miR-27a, H. pylori infection, and risk of gastric lesions in a chinese population. PLoS One 8: e61250. 23613822 10.1371/journal.pone.0061250 PMC3629121

[13] Lee HC, Kim JG, Chae YS, Sohn SK, Kang BW, et al (2010) Prognostic impact of microRNA-related gene polymorphisms on survival of patients with colorectal cancer. J Cancer Res Clin Oncol 136: 1073–1078. 20044760 10.1007/s00432-009-0754-6 PMC11828234

[14] Huang AJ, Yu KD, Li J, Fan L, Shao ZM (2012) Polymorphism rs4919510:C>G in mature sequence of human microRNA-608 contributes to the risk of HER2-positive breast cancer but not other subtypes. PLoS One 7: e35252. 22586447 10.1371/journal.pone.0035252 PMC3346742

[15] Liu T, Tang H, Lang Y, Liu M, Li X (2009) MicroRNA-27a functions as an oncogene in gastric adenocarcinoma by targeting prohibitin. Cancer Lett 273: 233–242. 18789835 10.1016/j.canlet.2008.08.003

[16] Sun Q, Gu H, Zeng Y, Xia Y, Wang Y, et al (2010) Hsa-mir-27a genetic variant contributes to gastric cancer susceptibility through affecting miR-27a and target gene expression. Cancer Sci 101: 2241–2247. 20666778 10.1111/j.1349-7006.2010.01667.x PMC11159034

[17] Liu Z, Xiao B, Tang B, Li B, Li N, et al (2010) Up-regulated microRNA-146a negatively modulate helicobacter pylori-induced inflammatory response in human gastric epithelial cells. Microbes Infect 12: 854–863. 20542134 10.1016/j.micinf.2010.06.002

[18] Hishida A, Matsuo K, Goto Y, Naito M, Wakai K, et al (2011) Combined effect of miR-146a rs2910164 G/C polymorphism and toll-like receptor 4 +3725 G/C polymorphism on the risk of severe gastric atrophy in Japanese. Dig Dis Sci 56: 1131–1137. 20721625 10.1007/s10620-010-1376-1

[19] Peng S, Kuang Z, Sheng C, Zhang Y, Xu H, et al (2010) Association of microRNA-196a-2 gene polymorphism with gastric cancer risk in a chinese population. Dig Dis Sci 55: 2288–2293. 19834808 10.1007/s10620-009-1007-x

[20] Wang S, Tao G, Wu D, Zhu H, Gao Y, et al (2013) A functional polymorphism in MIR196A2 is associated with risk and prognosis of gastric cancer. Mol Carcinog 52: 87–95. 10.1002/mc.22017 23423813

[21] Ahn DH, Rah H, Choi YK, Jeon YJ, Min KT, et al (2012) Association of the miR-146aC>G, miR-149T>C, miR-196a2T>C, and miR-499A>G polymorphisms with gastric cancer risk and survival in the korean population. Mol Carcinog 52: 39–51. 10.1002/mc.21962 23001871

[22] von Frowein J, Pagel P, Kappler R, von Schweinitz D, Roscher A, et al (2011) MicroRNA-492 is processed from the keratin 19 gene and up-regulated in metastatic hepatoblastoma. Hepatology 53: 833–842. 21319197 10.1002/hep.24125

[23] Gaedcke J, Grade M, Camps J, Sokilde R, Kaczkowski B, et al (2012) The rectal cancer microRNAome—microRNA expression in rectal cancer and matched normal mucosa. Clin Cancer Res 18: 4919–4930. 22850566 10.1158/1078-0432.CCR-12-0016 PMC6298229

[24] Kupcinskas L, Wex T, Kupcinskas J, Leja M, Ivanauskas A, et al (2010) Interleukin-1B and interleukin-1 receptor antagonist gene polymorphisms are not associated with premalignant gastric conditions: A combined haplotype analysis. Eur J Gastroenterol Hepatol 22: 1189–1195. 20631624 10.1097/MEG.0b013e32833cf3d5

[25] Kupcinskas J, Wex T, Bornschein J, Selgrad M, Leja M, et al (2011) Lack of association between gene polymorphisms of angiotensin converting enzyme, nod-like receptor 1, toll-like receptor 4, FAS/FASL and the presence of helicobacter pylori-induced premalignant gastric lesions and gastric cancer in caucasians. BMC Med Genet 12 112-2350-12-112. 10.1186/1471-2350-12-112 PMC3166912 21864388

[26] Dixon MF, Genta RM, Yardley JH, Correa P (1996) Classification and grading of gastritis. the updated sydney system. international workshop on the histopathology of gastritis, houston 1994. Am J Surg Pathol 20: 1161–1181. 8827022 10.1097/00000478-199610000-00001

[27] Uemura N, Okamoto S, Yamamoto S, Matsumura N, Yamaguchi S, et al (2001) Helicobacter pylori infection and the development of gastric cancer. N Engl J Med 345: 784–789. 11556297 10.1056/NEJMoa001999

[28] Lauren P (1965) The two histological main types of gastric carcinoma: Diffuse and so-called intestinal-type carcinoma: an attempt at a histo-clinical classification. Acta Pathol Microbiol Scand 64: 31–49. 14320675 10.1111/apm.1965.64.1.31

[29] Purcell S, Neale B, Todd-Brown K, Thomas L, Ferreira MA, et al (2007) PLINK: A tool set for whole-genome association and population-based linkage analyses. Am J Hum Genet 81: 559–575. 17701901 10.1086/519795 PMC1950838

[30] Zhou Y, Du WD, Chen G, Ruan J, Xu S, et al (2012) Association analysis of genetic variants in microRNA networks and gastric cancer risk in a chinese han population. J Cancer Res Clin Oncol 138: 939–945. 22350505 10.1007/s00432-012-1164-8 PMC11824628

[31] Xu Q, He CY, Liu JW, Yuan Y (2013) Pre-miR-27a rs895819A/G polymorphisms in cancer: A meta-analysis. PLoS One 8: e65208. 23762318 10.1371/journal.pone.0065208 PMC3676439

[32] Okubo M, Tahara T, Shibata T, Yamashita H, Nakamura M, et al (2010) Association between common genetic variants in pre-microRNAs and gastric cancer risk in japanese population. Helicobacter 15: 524–531. 21073609 10.1111/j.1523-5378.2010.00806.x

[33] Wang J, Bi J, Liu X, Li K, Di J, et al (2012) Has-miR-146a polymorphism (rs2910164) and cancer risk: A meta-analysis of 19 case-control studies. Mol Biol Rep 39: 4571–4579. 21947843 10.1007/s11033-011-1247-7

[34] Wang F, Sun G, Zou Y, Li Y, Hao L, et al (2012) Association of microRNA-499 rs3746444 polymorphism with cancer risk: Evidence from 7188 cases and 8548 controls. PLoS One 7: e45042. 22970328 10.1371/journal.pone.0045042 PMC3438197

[35] Zhang H, Su YL, Yu H, Qian BY (2012) Meta-analysis of the association between mir-196a-2 polymorphism and cancer susceptibility. Cancer Biol Med 9: 63–72. 23691458 10.3969/j.issn.2095-3941.2012.01.012 PMC3643645

[36] Yoon KA, Yoon H, Park S, Jang HJ, Zo JI, et al (2012) The prognostic impact of microRNA sequence polymorphisms on the recurrence of patients with completely resected non-small cell lung cancer. J Thorac Cardiovasc Surg 144: 794–807. 22818121 10.1016/j.jtcvs.2012.06.030

[37] Lin M, Gu J, Eng C, Ellis LM, Hildebrandt MA, et al (2012) Genetic polymorphisms in MicroRNA-related genes as predictors of clinical outcomes in colorectal adenocarcinoma patients. Clin Cancer Res 18: 3982–3991. 22661538 10.1158/1078-0432.CCR-11-2951 PMC4141857

[38] Ryan BM, McClary AC, Valeri N, Robinson D, Paone A, et al (2012) Rs4919510 in hsa-mir-608 is associated with outcome but not risk of colorectal cancer. PLoS One 7: e36306. 22606253 10.1371/journal.pone.0036306 PMC3350523

